# Laser capture microdissection as a method for investigating the human hair follicle microbiome reveals region-specific differences in the bacteriome profile

**DOI:** 10.1186/s13104-023-06302-5

**Published:** 2023-03-06

**Authors:** Marta B. Lousada, J Edelkamp, T Lachnit, M Fehrholz, F Jimenez, R Paus

**Affiliations:** 1 Monasterium Laboratory, Skin&Hair Research, Muenster, Germany; 2 Zoological Institute, Christian-Albrechts University Kiel, Kiel, Germany; 3 Mediteknia Skin & Hair Lab, Las Palmas de Gran Canaria, Spain; 4 Medical Pathology Group, IUIBS, Universidad de Las Palmas de Gran Canaria, Las Palmas, Spain; 5 Dr Phillip Frost Department of Dermatology & Cutaneous Surgery, University of Miami Miller School of Medicine, Miami, FL USA; 6 CUTANEON Skin & Hair Innovations, Hamburg, Germany

**Keywords:** Hair follicles, Microbiome, 16S rRNA sequencing

## Abstract

**Objective:**

Human hair follicles (HFs) are populated by a rich and diverse microbiome, traditionally evaluated by methods that inadvertently sample the skin microbiome and/or miss microbiota located in deeper HF regions. Thereby, these methods capture the human HF microbiome in a skewed and incomplete manner. This pilot study aimed to use laser-capture microdissection of human scalp HFs, coupled with 16S rRNA gene sequencing to sample the HF microbiome and overcome these methodological limitations.

**Results:**

HFs were laser-capture microdissected (LCM) into three anatomically distinct regions. All main known core HF bacterial colonisers, including *Cutibacterium*, *Corynebacterium* and *Staphylococcus*, were identified, in all three HF regions. Interestingly, region-specific variations in α-diversity and microbial abundance of the core microbiome genera and *Reyranella* were identified, suggestive of variations in microbiologically relevant microenvironment characteristics. This pilot study therefore shows that LCM-coupled with metagenomics is a powerful tool for analysing the microbiome of defined biological niches. Refining and complementing this method with broader metagenomic techniques will facilitate the mapping of dysbiotic events associated with HF diseases and targeted therapeutic interventions.

**Supplementary Information:**

The online version contains supplementary material available at 10.1186/s13104-023-06302-5.

## Introduction

Hair follicles (HFs) are a crucial component of human skin physiology and homeostasis [1–4]. These immune-privileged mini-organs are populated by a much richer microbiome than the human skin surface, whose microbiome is rapidly repopulated by the HF microbiome following skin disinfection [5, 6]. Furthermore, the role of HF microbiome dysbiosis in human skin physiology, regulation of the skin immune system, several non-infectious and infectious hair and skin diseases, has become a recent focus of interest in skin biology and dermatology [7–9]. Nevertheless, the exact composition of the HF-specific microbiome remains unclear and underexplored, due to limitations arising from the chosen microbiome sampling methods [5].

Typically, these methods include skin swabs, pore and tape strips, and cyanoacrylate gel biopsies [10–12]. Although these may suffice to characterise the resident skin microbiome communities, they fail to probe the deeper regions of the HF epithelium, completely miss the HF mesenchyme (Figure S1), and do not robustly distinguish between the HF and skin microbiomes [13, 14]. Likewise, plucked hair shafts leave crucial components of the HF epithelium and the entire HF mesenchyme unsampled [15, 16], and have a tendency to be confounded by a sampling bias towards assessing the microbiome associated with “quiescent” (telogen) HFs [17], since their short, loosely anchored club hair shafts are much easier to pluck than those of actively growing (anagen) HFs [18].

Previously, skin biopsies coupled with laser-capture microdissection (LCM) allowed the assessment of the spatial distribution of the subepidermal human microbiome [19]. This technology allows visualisation of the tissue or cells of interest under the microscope, selective laser-microdissection of the target area and transfer of the isolated sample into a separate container with laser pulse [20]. Hence, this hands-free microdissection method limits exposure of the samples to external sources and allows enrichment of areas of interest providing a higher degree of spatial resolution than the above-mentioned methods [21]. Therefore, this study aimed at the application of LCM technology to HF microbiome analysis, through 16 S rRNA gene sequencing, to overcome these limitations.

## Main text

## Materials and methods

### Sample collection

Occipital anagen VI follicular unit extractions (0.8-1 mm diameter mini-biopsies) were obtained from the scalp of three healthy volunteers, aged 45 (donor 1), 25 (donor 2), and 66 years (donor 3), undergoing routine hair transplantation surgery after informed, written participant consent and ethics committee approval from the Comité de Bioética de la Universidad Fernando Pessoa Canarias (03 (2020-06-22)) and the University of Muenster 2020-954-f-S. Following surgery, HFs were directly embedded in optimal cutting temperature compound. Samples were then cryo-sectioned into 10 μm sections, collected on MembraneSlide 1.0 PET (Zeiss), pre-treated as according to the manufacturer’s instructions, and stored at -80 °C.

### Laser-capture microdissection (LCM), DNA extraction and 16S rRNA gene sequencing

LCM procedure was performed on the following day using the contact-free PALM MicroBeam (Zeiss) with the PALM Robo software (Zeiss) [22]. Five HFs per donor were divided into three sections: an upper section, from the most distal point of the infundibulum to below the sebaceous gland duct; a middle section from this region to above the bulb, and a lower section, the hair bulb and dermal papilla (Figure S1). As technical replicates, subsections of each region were collected from different HF anatomical compartments. Following tissue isolation, DNA was extracted using the SmartExtract DNA kit (Eurogentec), following the manufacturer’s protocol, and precipitated with ethanol and carrier glycogen (ThermoFisher). Due to the low biomass of the samples, each compartment was pooled from five HFs per donor. Unlike previous studies [11, 12, 19], our method yielded enough DNA to omit a PCR amplification step. 16S rRNA gene (V1-V2) sequencing was performed at the University of Kiel in a pair-end modality on a Illumina NextSeq 500 platform rendering 2×150 bp pair-end sequences. Technical replicates were sequenced separately.

### Taxonomic annotation and statistical analysis

Following sequencing, sequence FASTA files were imported into Mothur v.1.44.3 and merged into a single file [23]. The most abundant sequence per cluster was considered a real biological sequence and assigned the count of all reads in the cluster, while the remainder of the reads were considered to contain errors as a product of sequencing. The representative reads from all clusters were subjected to chimera removal using the VSEARCH algorithm [24]. The remaining reads were aligned against a database of the target 16S rRNA gene sequences from version 138 of the SILVA database [25]. Relative abundance was determined by dividing each taxon count by the total number of filtered reads. Each sample was filtered to remove low abundant OTU clusters. All OTUs whose relative abundance was below 0.1% of the total number of filtered sequences in that sample were excluded. Percentage identities for phylum, family, and genus levels were of 97%. Low abundance OTUs were grouped in a single category termed “Others”, with a cut off at the top 20 most abundant taxa. Sequences are publicly available under accession number PRJNA849000, on the SRA database. Statistical analyses were performed in R (R version 4.0.4) [26], using the vegan [27] and tidyverse [28] packages. Similarity percentage (SIMPER) analysis was conducted to the estimate the dissimilarity between the HF regions analysed and performed with Primer 7, Version 7.0.17 [29].

## Results and discussion

**Fig. 1:**
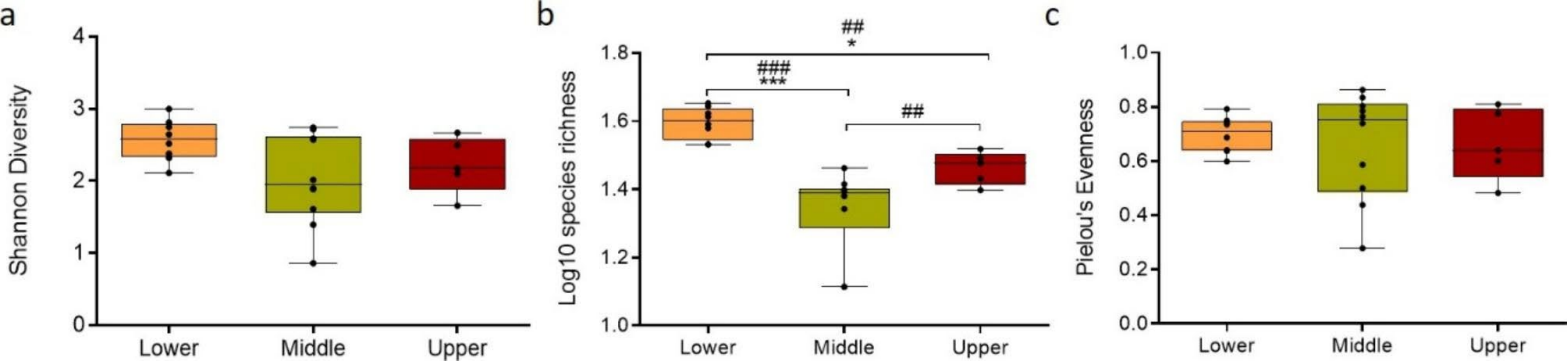
**Diversity indexes of the bacterial communities of the HF. (a)** Alpha-diversity per samples from the lower, middle, and upper HF regions, as depicted by the Shannon’s alpha-diversity. **(b)** Species richness and **(c)** Pielou’s eveness index. Mean ± SEM, from 3 independent donors (n = 3 HFs per region), D’Agostino & Pearson omnibus normality test, no Gaussian distribution, Kruskal-Wallis test (p = 0.0982 [Shannon diversity], p < 0.001 [species richness] and p = 0.9492 [Pielou’s evenness]), Tukey’s multiple comparisons test, *p < 0.05, ***p < 0.01, and Mann–Whitney test, ##p < 0.01 and ###p < 0.001

**Fig. 2:**
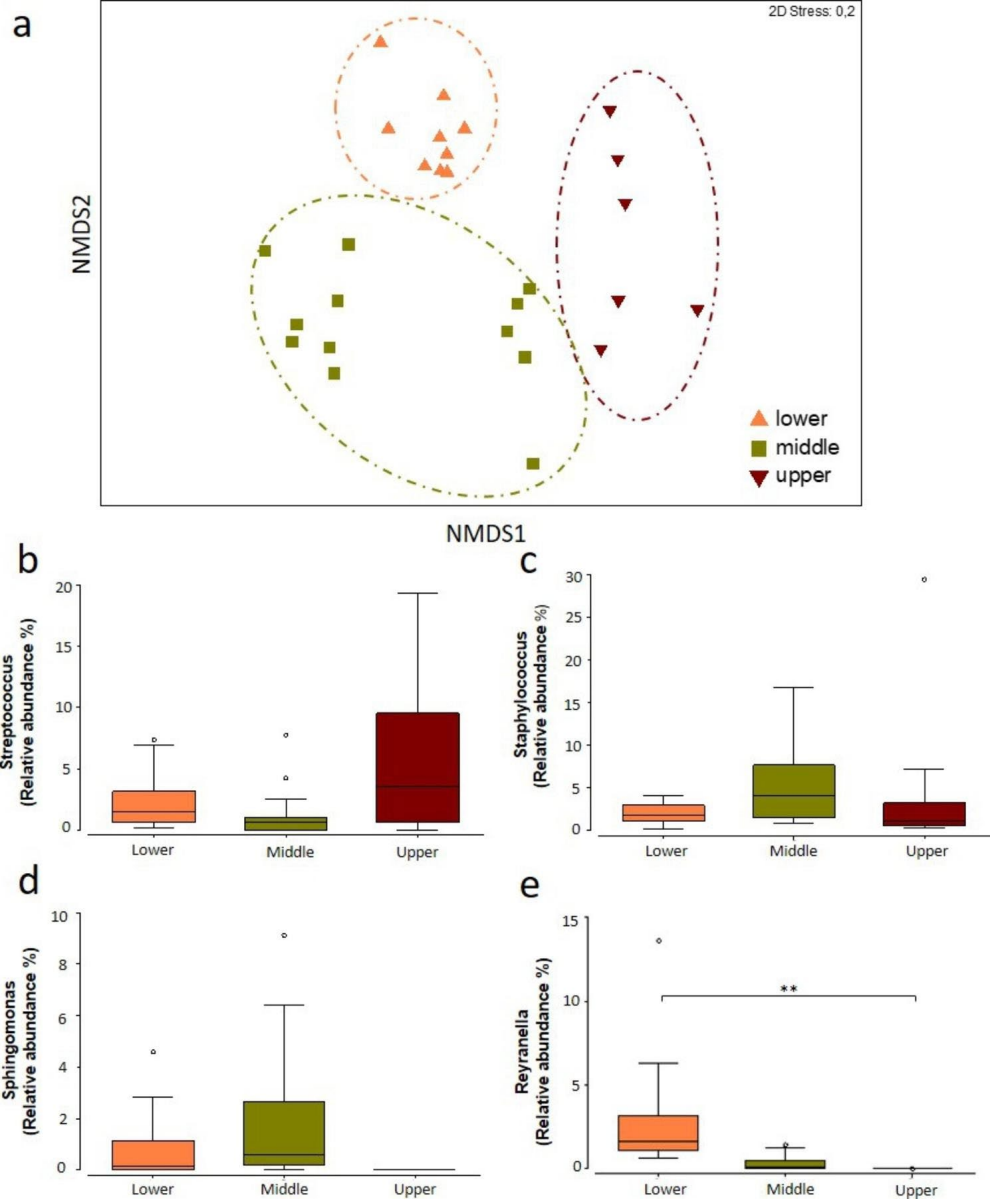
**Microbial community variation over the different HF regions. (a) **Non-metric multidimensional scaling (NMDS) plot for beta-diversity patterns based on Bray-Curtis distances from the three HF regions. Numbers refer to the different donors: (1) 45 years old male; (2) 25 years old male, and (3) 66 years old male. NMDS stress value= 0.2. **(b-e)** Percentage of abundance of the main contributors towards community variations between HF regions, according to SIMPER analysis. Mean abundance is the mean value of the relative abundance of each genus in the sampling groups (ANOVA test, **p< 0.01)

To analyse the bacterial composition of human scalp HFs, full-length HFs that had been microdissected free of all extrafollicular tissue (26,30,31), were laser capture-microdissected into lower, middle and upper portions, corresponding to anatomically well-defined compartments at distinct levels of the human HF epithelium and mesenchyme (Figure S1), and respective DNA was extracted and analysed by 16S rRNA gene sequencing (19,32,33). A total of 93,118 to 108,008 high-quality reads were obtained from each of the different analysed regions (data not shown). As a measure of alpha-diversity, the Shannon diversity index [30] was calculated. Variances were found between the lower and middle regions (Shannon diversity index: p = 0.0982; Fig. 1a), with the lower HF section (hair bulb and dermal papilla, Figure S1) registering a tendentially higher diversity profile compared to the other regions (Fig. 1a). These therefore suggest that the upper HF communities might be dominated by a few main taxa, which limit the growth of other lower abundance commensals (Figs. 1a-b and 2). In line with this, while no significant compositional differences were observed between the three HF regions, there were visible changes in the percentage of abundance of each taxa depending on the HF region, with Pielou’s evenness [30], meaning the similarity of frequency of the identified genera, being higher in the middle and lower HF regions (Fig. 1c). The microbiome differences between the selected HF regions were further assessed by non-metric multidimensional scaling (NMDS) analysis based on Bray-Curtis similarity distances [31]. In line with previous reports, all biological replicates clustered according to the region of collection, suggesting that each HF region is characterised by a specific microbial colonisation profile (Fig. 2a) [10]. This was confirmed by perMANOVA test [31], based on the same similarity index (lower vs. middle p < 0.05, lower vs. upper p < 0.05 and middle vs. upper p < 0.01).

Further, as expected, sample clustering revealed both inter- and intra-individual variability of the communities in the different HF regions. Interestingly, among the most relevant cluster drivers, *Streptococcus, Staphylococcus*, and *Sphingomonas* were identified, driving the upper, middle and lower HF regions, respectively (Fig. 2b-d). These microbiome variations could therefore reflect the compartmental variations in nutritional availability, antimicrobial peptide production, and/or human HF immune system components within the HF micro-environments [32], which favour the growth of selected bacteria. Indeed, the HF exhibits marked compartmental differences, for example, in the availability of nutrients, metabolites, antimicrobial peptides, and immunological characteristics [33–37]. Due to sebum production and secretion by the sebaceous gland, the suprainfundibular portion of the distal outer root sheath (see infundibulum, Figure S1) is rich in triglycerides, which favour the growth of known hydrolysers such as *Staphylococcus*, while the proximity to the stratum corneum, provides a source of ceramides, cholesterol and filaggrin that favour the growth of other lipid degraders, such as *Streptococcus* [14, 38, 39]. These nutrient sources may drive the establishment of these bacteria in the distal HF regions. Thereby, a conceivable explanation is that the upper HF environment favours the growth of these biofilm-forming commensals, which prevent the growth of the less numerous bacteria, which in turn are favoured in the lower HF. However, a confounding role of skin disinfection prior to skin biopsy, which primarily depletes the most distally located microbial communities of the upper HF, cannot be excluded.

**Fig. 3:**
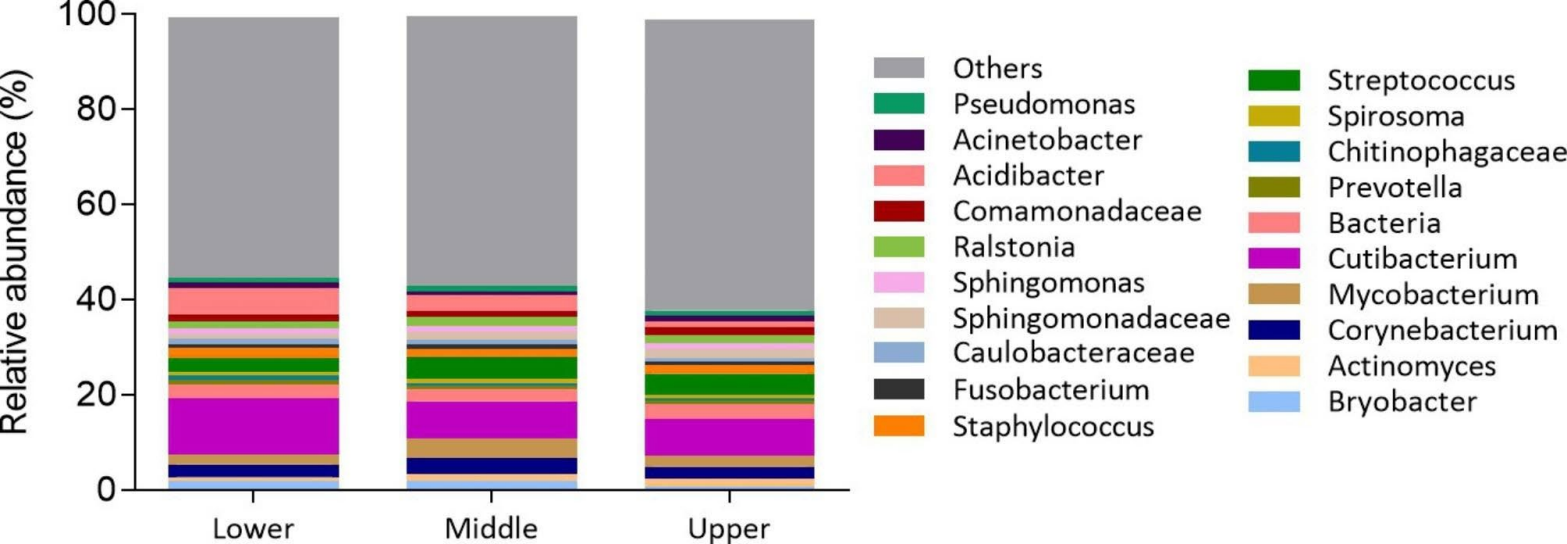
**Relative abundance of the top 20 bacterial communities within the HF.** Relative abundance of the main 20 bacteria taxa resident in the lower, middle and upper portions of the HF. Results are presented as the percentage (%) of the total sequences. All OTUs whose relative abundance was below 0.1% of the total number of filtered sequences in that sample were excluded

Additional analysis of microbial composition, showed that, consistent with the phyla profile of sebaceous sites, Proteobacteria and Actinobacteria were the predominant phyla found across all three regions, followed by Firmicutes and Bacteroides (Figure S2) [40]. In the lower portion of the HF, about 36.5% and 27.1% of the sequences were assigned to Proteobacteria and Actinobacteria, respectively. Comparably, in the middle and upper HF regions 35.2% and 36.1% of the reads assigned to Proteobacteria and 24.3% and 23.6% to Actinobacteria, respectively. Similarly, analysis at genera level demonstrated a predominance of *Cutibacterium*, *Streptococcus* and *Corynebacterium* in all regions of the HF, as previously described by others [10–12] (Figs. 2 and 3). However, while previous works reported these genera as the most abundant in the HF, comprising between 60 and 90% of the total sequences [11, 12, 14], LCM-based 16 S rRNA sequencing revealed a lower abundance of these microbes in all three HF regions (Fig. 3).

Likewise, other previously reported less abundant genera were identified with slight variations in their relative abundances (Fig. 3), attesting to the sensitivity and specificity of LCM-based 16S rRNA gene sequencing for the purpose of human HF microbiome analysis. Particularly, the lower HF registered a tendentially higher abundance of *Cutibacterium* (11.8%) and a lower percentage of *Streptococcus* (3.0%) and *Staphylococcus* (2.2%), when compared to the middle (7.8%, 4.8%, and 1.4%, respectively) and upper (7.8%, 4.4%, and 1.8%, respectively) portions of the HF (Figs. 2 and 3). Further, LCM-based 16S rRNA gene sequencing also identified bacterial genera that, to our knowledge, have not been previously described to populate the human HF, namely *Acidibacter*, *Fusobacterium*, and *Bryobacter* (Fig. 3). Although these differences would require further investigation in more donors, given the high inter-individual differences of the human microbiome, these variations could derive from the differential biological starting material, including the investigated patient populations and/or the sampling method. Notably, contrarily to other studies, the present study is the first to dissociate the HF from the epidermis, dermis, sebaceous and eccrine glands and the hair shaft (Figure S1).

Additionally, although for this proof-of-principle study, broader HF regions were examined in order to overcome the challenges arising from below-threshold tissue sample size, LCM of sharply defined, anatomically distinct compartments, such as the epithelial and mesenchymal compartments of the hair bulb was also possible (Figure S3). Interestingly, this comparative analysis revealed a higher abundance of *Staphylococcus* and *Cutibacterium* in the mesenchyme compared to the epithelium (6.9% vs. 0.8% and 37.4% vs. 24.5%, respectively) (Figure S3). These compartmental variations found in the HF become of special interest in the context of HF diseases characterised by different anatomical target areas of the HF. For instance, both acne vulgaris and hidradenitis suppurativa primarily affect the infundibulum, while alopecia areata exclusively affects the bulb region (Figure S1) [41, 42].

Taken together, this pilot study shows that LCM technology coupled with metagenomics is a powerful tool for analysing biological niches and their microbiome. This method provides researchers with a higher-resolution tool to understand the impact of HF commensals on human HF biology and the role of dysbiosis on folliculitis and hair loss disorders. In the future, this technique can be redefined by complementing 16S rRNA gene sequencing with RNA* in situ* hybridisation for selected bacteria of interest [19] and techniques aimed at increasing the measurement of viable bacteria [43]. Together these techniques will provide a detailed map of the HF microbiome and facilitate the development of targeted human HF microbiome manipulation, through pre- and pro-biotics, nutraceuticals and lentibiotics.

## Limitations

This study is limited by its small sample size, which may affect evaluations of the microbiome given the high inter-individual variability associated with microbiome studies, especially given the age variation and limited donor information in this cohort. Larger prospective studies are therefore needed.

## Electronic supplementary material

Below is the link to the electronic supplementary material.

Supplementary Material 1

## Data Availability

The datasets used and/or analysed during the current study are available from the corresponding author on reasonable request. Sequencing data was made publicly available under the accession number PRJNA849000 on the SRA database.

## Abbreviations

HF: Hair follicle
LCM: Laser-capture microdissection
