# Supplementary material for: Laser capture microdissection as a method for investigating the human hair follicle microbiome reveals region-specific differences in the bacteriome profile
Source: BMC Res Notes. 2023 Mar 6;16:29. doi: 10.1186/s13104-023-06302-5 (PMC9987047; doi:10.1186/s13104-023-06302-5)
Supplement: Supplementary file 1 — Supplementary Material 1 [file 13104_2023_6302_MOESM1_ESM.docx]

**Supplementary material**

**
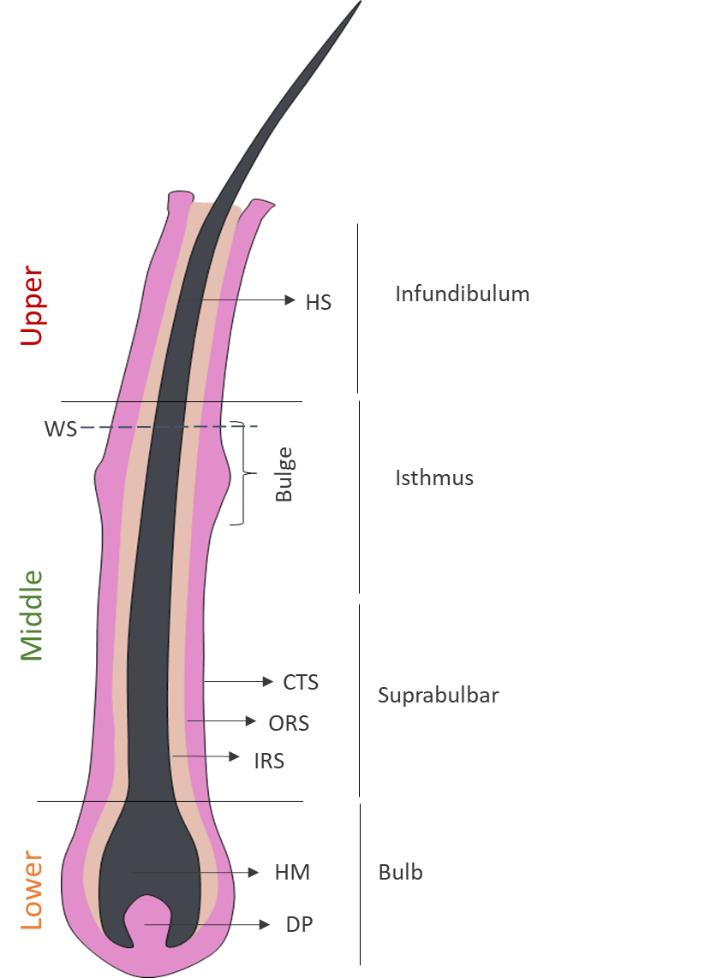
**

**Figure S1. Representation of the division of the hair follicle into lower, upper and middle portions evaluated.** The upper section started from the most distal point of the infundibulum to below the sebaceous gland duct; the middle section from above the bulb encompassing both isthmus (with bulge area of HF stem cells) and suprabulbar regions, and the lower section encompassing the proximal bulb, including the dermal papilla (DP). Two main tissue lineages are found in the hair follicle, the mesenchyme, which comprises the innermost HF layers, including the inner and outer root sheaths (IRS and ORS) and hair matrix (HM), and the epithelium, *i.e.,* the surrounding connective tissue sheath (CTS), which within the bulb region is also termed dermal sheath, and the DP [2]. From the bulge region of stem cells downwards (WS), the HF exhibits immune-privileged characteristics, with downregulation of MHC class I molecules, expression of immuno-suppressants and of antigen-presenting cells [44]. Note that all analysed HFs were microdissected as indicated in the figure and were free of any extrafollicular tissue (including epidermal remnants, adipocytes and interfollicular dermis). WS: immunological “watershed”; HS: hair shaft.

**Figure S2. Taxonomic relative abundance of the main bacterial phyla identified in the lower, middle, and upper portions of the HF.** Results are presented as the percentage of total sequences.

**Figure S3. Taxonomic relative abundance of the 20 main bacterial genera populating the HF bulb.**  Relative abundance of the main 20 bacteria taxa resident in the mesenchymal (hair matrix) and epithelial (dermal cup and dermal papilla) portions of the HF bulb. Results are presented as the percentage of the total sequences. All OTUs whose relative abundance was below 0.1% of the total number of filtered sequences in that sample were excluded.
